# Transcriptome analysis reveals the molecular mechanisms of heterosis on thermal resistance in hybrid abalone

**DOI:** 10.1186/s12864-021-07954-y

**Published:** 2021-09-08

**Authors:** Qizhen Xiao, Zekun Huang, Yawei Shen, Yang Gan, Yi Wang, Shihai Gong, Yisha Lu, Xuan Luo, Weiwei You, Caihuan Ke

**Affiliations:** 1 State Key Laboratory of Marine Environmental Science, College of Ocean and Earth Sciences, Xiamen University, Xiamen, 361102 People’s Republic of China; 2 Fujian Key Laboratory of Genetics and Breeding of Marine Organisms, Xiamen University, Xiamen, 361102 People’s Republic of China

**Keywords:** Heterosis, Abalone, Heat stress, Transcriptome, Alternative splicing, Non-additive expression

## Abstract

**Background:**

Heterosis has been exploited for decades in different animals and crops due to it resulting in dramatic increases in yield and adaptability. Hybridization is a classical breeding method that can effectively improve the genetic characteristics of organisms through heterosis. Abalone has become an increasingly economically important aquaculture resource with high commercial value. However, due to changing climate, abalone is now facing serious threats of high temperature in summer. Interspecific hybrid abalone (*Haliotis gigantea* ♀ × *H. discus hannai* ♂, SD) has been cultured at large scale in southern China and has been shown high survival rates under heat stress in summer. Therefore, SD has become a good model material for heterosis research, but the molecular basis of heterosis remains elusive.

**Results:**

Heterosis in thermal tolerance of SD was verified through Arrhenius break temperatures (ABT) of cardiac performance in this study. Then RNA-Sequencing was conducted to obtain gene expression patterns and alternative splicing events at control temperature (20 °C) and heat stress temperature (30 °C). A total of 356 (317 genes), 476 (435genes), and 876 (726 genes) significantly diverged alternative splicing events were identified in *H. discus hannai* (DD), *H. gigantea* (SS), and SD in response to heat stress, respectively. In the heat stress groups, 93.37% (20,512 of 21,969) of the expressed genes showed non-additive expression patterns, and over-dominance expression patterns of genes account for the highest proportion (40.15%). KEGG pathway enrichment analysis showed that the overlapping genes among common DEGs and NAGs were significantly enriched in protein processing in the endoplasmic reticulum, mitophagy, and NF-*κ*B signaling pathway. In addition, we found that among these overlap genes, 39 genes had undergone alternative splicing events in SD. These pathways and genes may play an important role in the thermal resistance of hybrid abalone.

**Conclusion:**

More alternative splicing events and non-additive expressed genes were detected in hybrid under heat stress and this may contribute to its thermal heterosis. These results might provide clues as to how hybrid abalone has a better physiological regulation ability than its parents under heat stress, to increase our understanding of heterosis in abalone.

## Background

Heterosis, or hybrid vigor, refers to the phenomenon in which hybrid offspring surpass their parents in the desired character [1]. A batch of hybrid livestock (e.g., pig) and crops (e.g., rice) have shown better performance in growth and environmental adaptation when compared with their parents, therefore it has received extensive research [2]. Various genetic models have been put forward to explain heterosis, including dominance, overdominance, and epistatic hypothesis [3]. Recently, the overdominance hypothesis has been supported by a lot of experimental research [4–6], in which non-additive effects are described as a consequence of genetic differences between the homozygous parents and their heterozygous hybrids [7]. The discussion of the genetic basis of heterosis has lasted for nearly a century, but that of the molecular mechanism of heterosis still remain elusive Next-generation sequencing (NGS) technologies offer the potential to uncover the molecular mechanism of heterosis at the transcriptional level [8]. The identification of non-additive genes is accomplished based on their expression patterns which may finally shape complex traits of hybrid organisms. Genome-wide changes in gene expression have been documented in hybrids of maize [9], rice [10], soybean [11], wheat [12], cotton [13], yellow catfish [14], pearl oyster [15], pufferfish [16], sea cucumber [17] and black seabream [18]. Transcriptomic analysis provides an efficient way to explore heterosis, and its results mainly include abundant differential expressed genes and alternative splicing (AS) events. AS refers to the regulatory processes to produce variably spliced mRNAs by selecting various combinations of splice sites within a pre-mRNA in eukaryotes. In humans, approximately 98% of multi-exonic genes are alternatively spliced, and alternative splicing producce diverse transcripts and proteins [19]. AS can significantly impact the transcriptome and proteome by creating multiple isoforms that can maintain the diversity of protein in eukaryotes [20]. Five AS events types have been recognized in animals, including skipped exons (SE), alternative 5’splice sites (A5SS), alternative 3’splice sites (A3SS), retained introns (RI), and mutually exclusive exons (MXE) [21]. As a post-transcriptional regulation process modulating gene expression, AS has been reported to play an important role in heterosis establishment [22].

Abalone has become an increasingly economically important aquaculture resource with high commercial value. However, ocean warming is predicted to greatly affect marine ecosystems [23], this seriously affected the abalone farming industry. Elevated temperature can increase oxygen consumption of aquatic animals, largely influencing the most metabolic processes in ectotherms [24]. Harmful end-products, such as reactive oxygen species (ROS), would also be produced and cumulated under heat stress [25]. In recent years, the abalone farming industry has experienced a notable expansion in China, whose yield accounting for more than 90% of the global aquaculture production (FAO, 2019). The Pacific abalone *Haliotis discus hannai* (DD), the main aquaculture abalone species in China, is naturally distributed in temperate water [26]. Due to the natural climate conditions in Fujian Province (the main abalone producing area in China), Pacific abalone is now facing serious threats of high temperature in summer. This pattern is exacerbated by increasingly serious global climate change [23]. To resolve this problem, new abalone species that can withstand high temperatures were introduced to China. The Xishi abalone *H. gigantea* (SS), which is naturally distributed along the coasts of Japan, has a wide range of temperature adaptability [27]. The hybrid *H. gigantea* ♀ × *H. discus hannai* ♂ (SD) was produced on large-scale and has been approved to exhibit heterosis in growth rate, survival rate, sub-low salinity adaptability, and thermal resistance [27–29]. Therefore, SD is a good model for heterosis research, the molecular basis of which remains elusive.

The objective of this study is to uncover the molecular mechanisms underlying the high superiority of temperature resistance in an interspecific hybrid. Thermal tolerance of hybrid (SD) and its parents (SS and DD) was first assessed through ABT measurements, to validate the heterosis of SD at physiological levels. Then RNA-Sequencing (RNA-Seq) was conducted to obtain gene expression patterns and alternative splicing events. These results might provide clues as to how hybrid abalone has a better physiological regulation ability than its parents under thermal stress, which also increase our understanding of heterosis in abalone.

## Results

### The comparison of growth traits

**Fig. 1:**
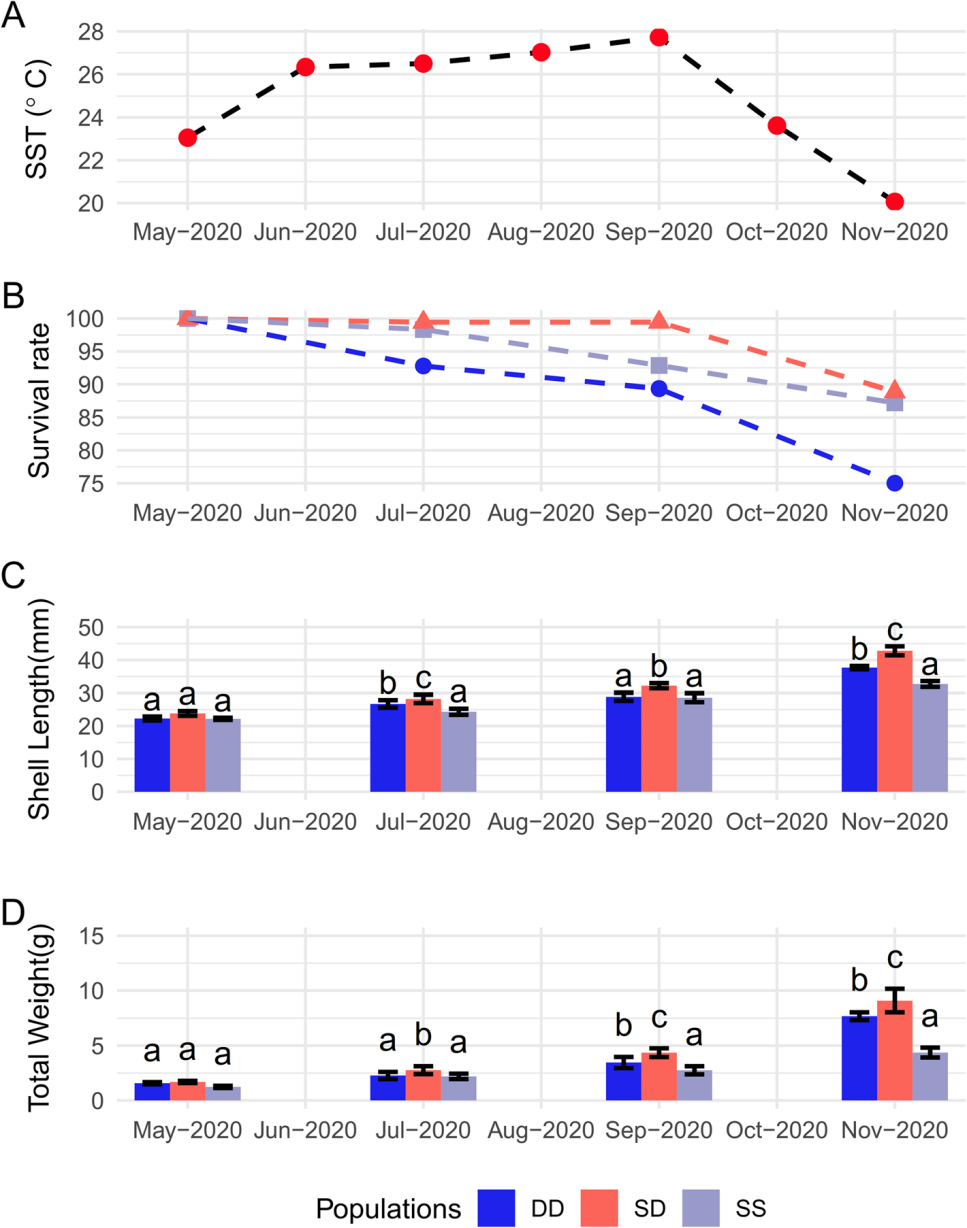
(**A**) The seawater surface temperature. (**B**) The survival rate of three abalone populations from May 2020 to Nov 2020. (**C**) The shell length of three abalone populations from May 2020 to Nov 2020. (**D**) The total weight of three abalone populations from May 2020 to Nov 2020. Different letters represent the significant difference among three populations (*P* < 0.05)

At the beginning of the breeding experiment, the three populations were not significantly different (*P* > 0.05) in shell length and total weight. After seven months, the shell lengths of DD, SS and SD abalones were 37.69 ± 0.51, 32.72 ± 0.89, and 42.80 ± 1.37 mm, respectively. The average weights of DD, SS and SD abalones were 7.69 ± 0.35, 4.37 ± 0.45, and 9.10 ± 1.06 g, respectively. The shell length and total weight of SD abalone were significantly higher than its parents (*P* < 0.05). The survival rate of SD was higher than that of DD and SS during high-temperature months (July to September) (Fig. 1).

### ABT measurement of cardiac performance

**Fig. 2:**
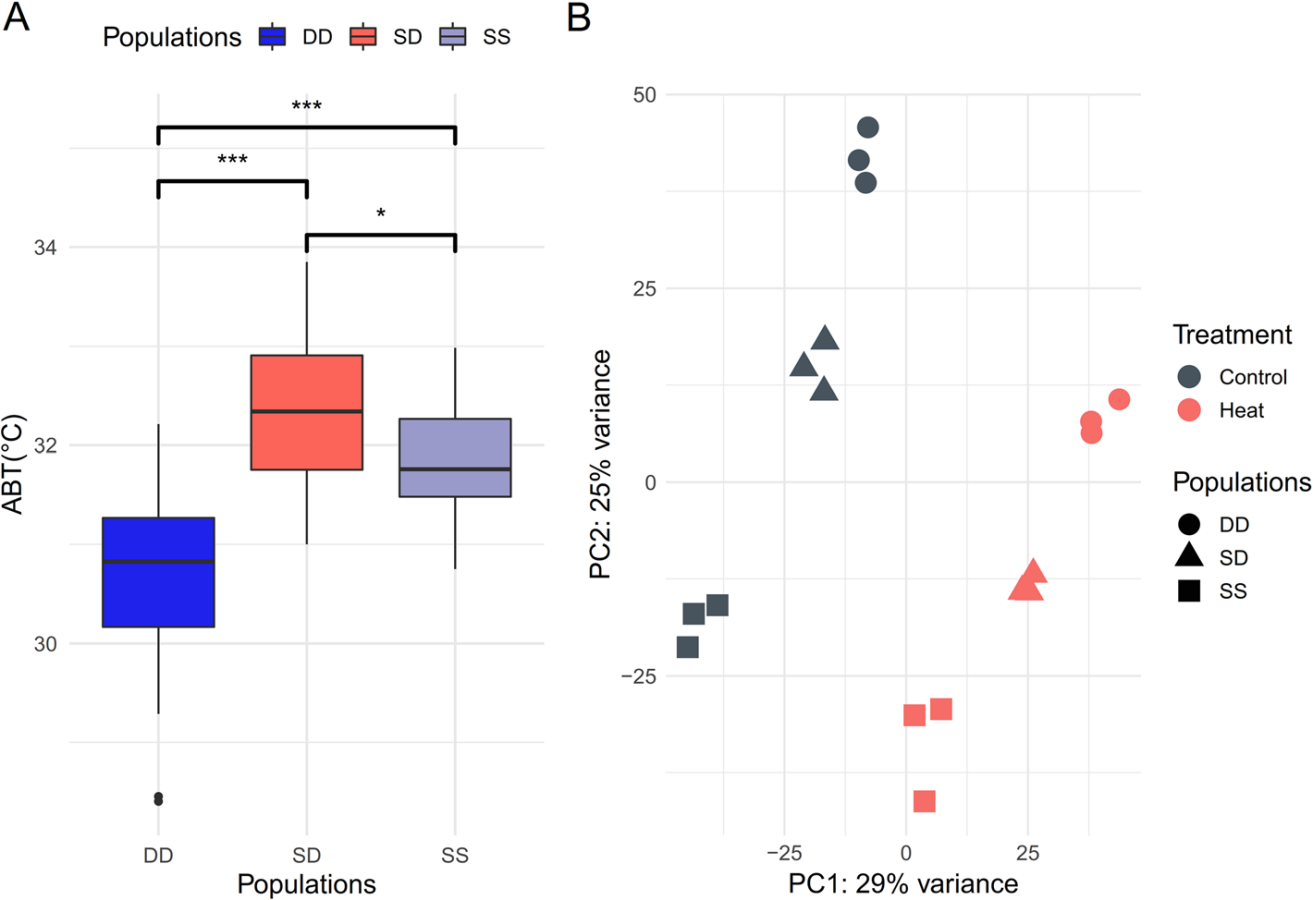
(**A**) The comparisons of thermal tolerance in three abalone populations base on ABT. ∗indicates *P* < 0.05; ∗∗∗indicates *P* < 0.01. (**B**) Principal component analysis plot based on the whole genome gene expression profiles

ABTs of DD, SS and SD were 30.66 ± 0.8 °C, 31.87 ± 0.59 °C and 32.38 ± 0.71 °C, respectively (Fig. 2A). ABTs of SD were significantly higher than those of DD and SS (*P* < 0.05). The hybrid abalone SD exhibited the best thermal tolerance, while DD was shown to be most sensitive to heat stress, indicating the heterosis of thermal resistance in hybrid SD.

### Transcriptome sequencing and identification of DEGs

In total, an average of 43.65 million raw reads per specimen (40.89–47.38 million raw reads) were obtained through RNA-sequencing. Then 39.22–44.99 million clean reads were filtered from each sequencing sample, while 71.66–88.11% of the reads were aligned to the reference genome. A principal component analysis (PCA) based on the whole genome gene expression profiles showed that six groups (3 populations × 2 treatment groups) clearly separated in the PC1 × PC2 score plot (Fig. 2B), with PC1 explaining 29% and PC2 explaining 25% of the total variance.

**Fig. 3:**
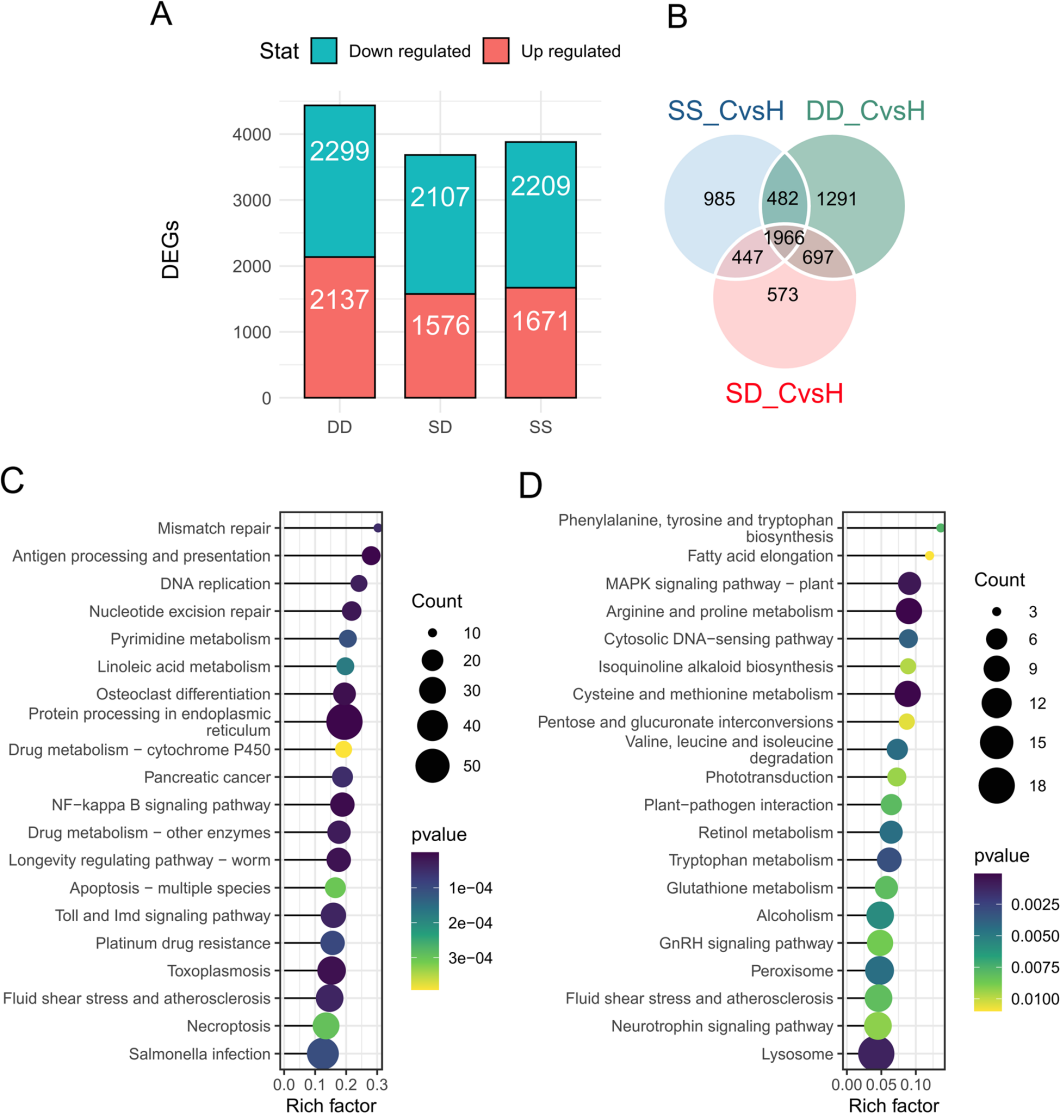
(**A**) Up- and down-regulated DEGs in three populations. (**B**) The Venn diagram of significant differently expressed genes (DEGs) in three abalone populations. DD_CvsH: the DEGs between the control group and heat stress group in DD. SS_CvsH: the DEGs between the control group and heat stress group in SS. SD_CvsH: the DEGs between the control group and heat stress group in SD. (**C**) Top 20 KEGG enrichment pathways of the common DEGs shared by SD, SS, and DD. (**D**) Top 20 KEGG enrichment pathways of the specifically expressed DEGs in SD compared to the SS and DD

Between the C and H groups, a total of 3880 DEGs in SS (referred to as “SS_CvsH”), 4436 DEGs in DD (referred to as “DD_CvsH”), and 3713 DEGs in SD (referred to as “SD_CvsH”) (Fig. 3B) were identified, with 1966 DEGs common to all three comparisons. Among these DEGs, 573, 1291, and 985 genes were specifically expressed in SD, DD, and SS. In addition, the numbers of overlap DEGs between SD_CvsH and DD_CvsH, SD_CvsH and SS_CvsH, and DD_CvsH and SS_CvsH were 2663, 2413, and 2448, respectively. Among the DEGs, the number of down-regulated genes was higher than the number of up-regulated genes (Fig. 3A). The results of KEGG pathway enrichment analysis showed that those 1966 common DEGs in three populations were enriched in the following pathways: protein processing in endoplasmic reticulum (57 genes), NF-*κ*B signaling pathway (25 genes), antigen processing and presentation (16 genes), osteoclast differentiation (22 genes), Toll and Imd signaling pathway (27 genes), fluid shear stress and atherosclerosis (32 genes), necroptosis (30 genes), and apoptosis (19 genes) (Fig. 3C). And these 573 specifically expressed DEGs in SD enriched in the following pathways: arginine and proline metabolism (9 genes), MAPK signaling pathway (7 genes), lysosome (18 genes), tryptophan metabolism (8 genes), cysteine and methionine metabolism (9 genes), glutathione metabolism (7 genes) (Fig. 3D).

The interaction analysis between population and environment was carried out using R DESeq2 package with *p*adj < 0.05 and |log2FoldChange| > 1 as the significance threshold to estimate the interaction effect on gene expression. The results showed that there were 143 significantly different genes in the comparison of “(heat SS-control SS) - (heat SD-control SD)”, and 26 significantly different genes in the comparison of “(heat DD-control DD) - (heat SD-control SD)”, and 236 significantly different genes in the comparison of “(heat SS-control SS) - (heat DD-control DD)”. There were a total of 261 DEGs influenced by interaction effects from population and environment. These genes were related to endocrine resistance, apoptosis, Toll and Imd signaling pathway, MAPK signaling pathway, and cytokine-cytokine receptor interaction.

### Expression patterns of non-additive genes

**Fig. 4:**
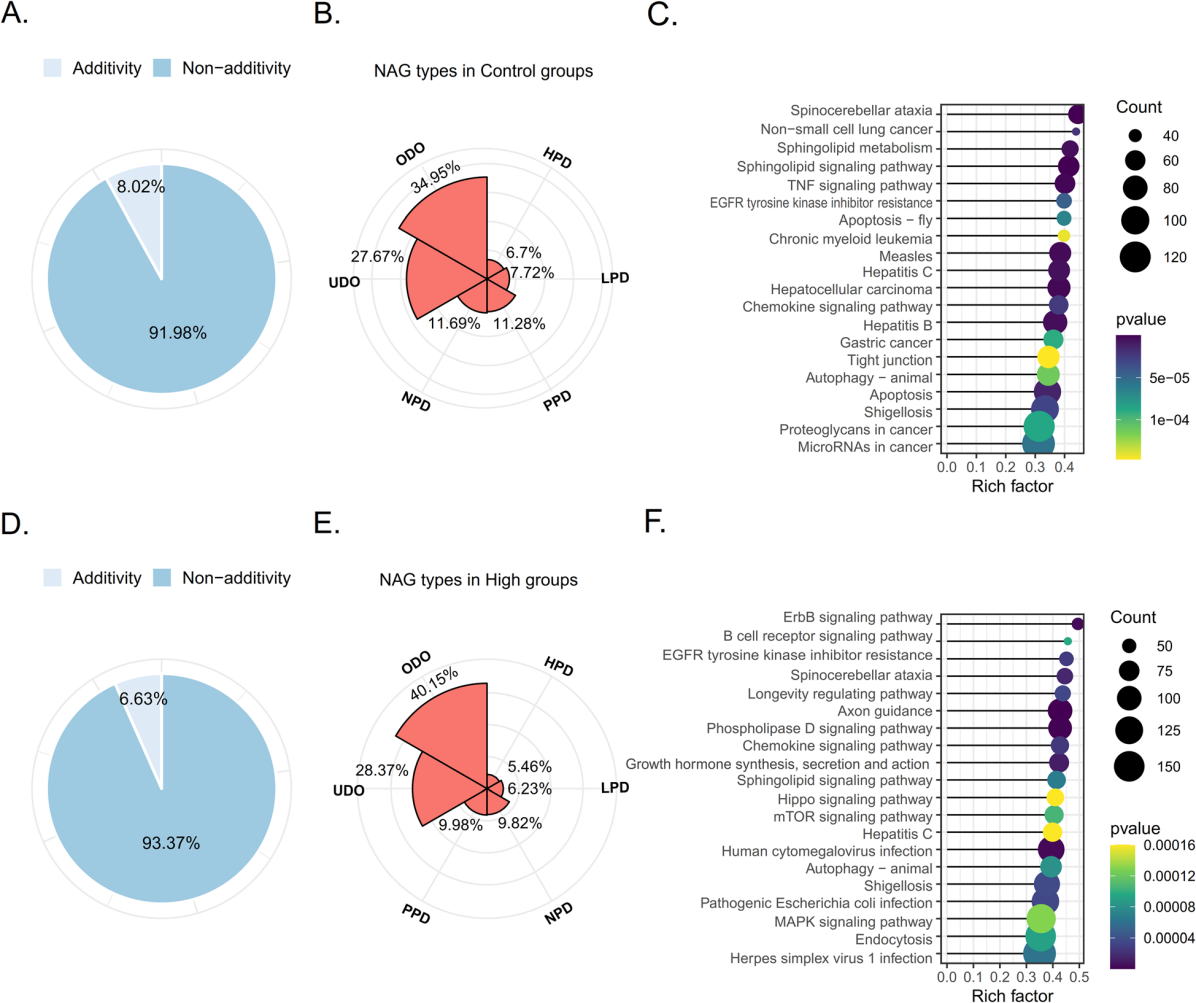
(**A**) The proportion of additivity and non-additivity genes in control groups. (**B**) The proportion of expression pattern for non-additively genes in control groups. (**C**) Top 20 KEGG enrichment pathways of the over-dominance in control group. (**D**) The proportion of additive and non-additive genes in heat stress groups. (**E**) The proportion of expression pattern for non-additively genes in heat stress group. (**F**) Top 20 KEGG enrichment pathways of the over-dominance in heat stress group. HPD: high-parent dominance, LPD: low-parent dominance, UDO: under-dominance, ODO: over-dominance, NPD: negative partial-dominance, PPD: positive partial-dominance

A total of 91.98% (20,206 of 21,969) of the genes detected in the C groups of three populations showed non-additive expression patterns (Fig. 4A), classified into six distinct classes: high-parent dominance (HPD), low-parent dominance (LPD), under-dominance (UDO), over-dominance (ODO), negative partial-dominance (NPD), and positive partial-dominance (PPD). Of 20,206 genes, 1354 (6.70%), 1559(7.72%), 7061 (34.94%), 5590 (27.67%), 2280 (11.28%) and 2362(11.69%) showed HPD, LPD, ODO, UDO, PPD, and NPD, respectively. ODO and UDO accounted for more than half of non-additive genes (Fig. 4B). The results of KEGG enrichment pathway analysis showed that 7061 ODO genes enriched in the following pathways: sphingolipid signaling pathway (65 genes), TNF signaling pathway (61 genes), apoptosis (95), chemokine signaling pathway (57 genes), EGFR tyrosine kinase inhibitor resistance (45 genes), and autophagy (71 genes) (Fig. 4C). The results of KEGG enrichment pathway analysis showed that those 5590 UDO genes enriched in the following pathways: oxidative phosphorylation (71 genes), thermogenesis (114 genes), citrate cycle (TCA cycle) (27 genes), protein processing in endoplasmic reticulum (84 genes), glycolysis/gluconeogenesis (25 genes), basal transcription factors (23 genes), and glucagon signaling pathway (46 genes).

A total of 93.37% (20,512 of 21,969) of the genes detected in the H groups of three populations showed non-additive expression patterns (Fig. 4D). Of 20,512 genes, 1119 (5.46%), 1278 (6.23%), 8235 (40.15%), 5819 (28.37%), 2047 (9.98%) and 2014 (9.82%) showed HPD, LPD, ODO, UDO, PPD, and NPD, respectively. ODO account for the highest proportion, more than 40% of non-additive genes (Fig. 4E). The results of KEGG enrichment pathway analysis showed that 8235 ODO genes enriched in the following pathways: phospholipase D signaling pathway (94 genes), axon guidance (100 genes), growth hormone synthesis, secretion and action (72 genes), chemokine signaling pathway (64 genes), EGFR tyrosine kinase inhibitor resistance (51 genes), autophagy (149 genes), B cell receptor signaling pathway (42 genes), and mTOR signaling pathway (68 genes) (Fig. 4F). The results of KEGG enrichment pathway analysis showed that 5819 UDO genes enriched in the following pathways: ribosome (117 genes), oxidative phosphorylation (80 genes), metabolism of xenobiotics by cytochrome P450 (40 genes), thermogenesis (118 genes) RNA transport (98 genes), and ribosome biogenesis in eukaryotes (61 genes).

### Identification of AS events

**Fig. 5:**
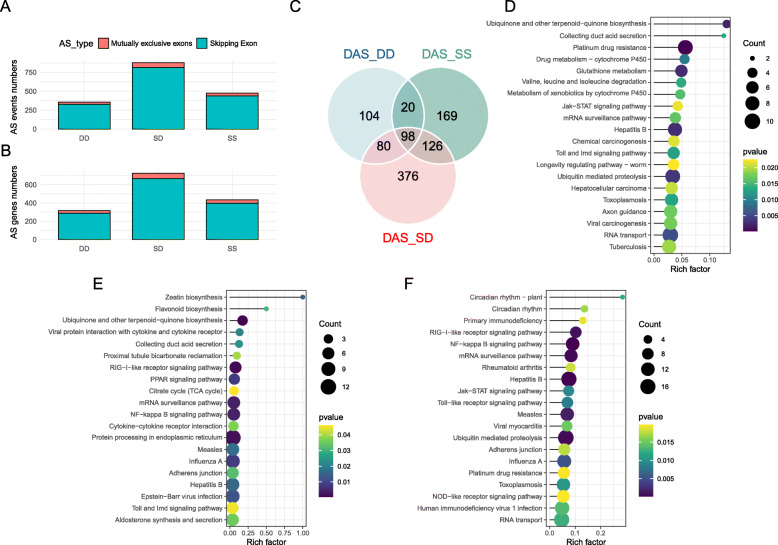
(**A**) Number of AS events type discovered among different populations. (**B**) Number of AS genes type discovered among different populations. (**C**) The Venn diagram of AS genes (DAS) in three abalone populations. (**D**) Top 20 KEGG enrichment pathways of the DAS in DD. (**E**) Top 20 KEGG enrichment pathways of the DAS in SS. (**F**) Top 20 KEGG enrichment pathways of the DAS in SD

The divergence of AS events between the C and H group among three populations were characterized. For a total of 356 (317 genes), 476 (435 genes), and 876 (726 genes) significant divergence AS events were identified in DD, SS, and SD, respectively (Fig. 5A–5C). The hybrid SD exhibited the most dramatic AS events and genes in response to heat stress, comparing with its parent SS and DD species. These divergence AS events included skipping exons (90.03–91.73%) and mutually exclusive exons (8.27–9.97%). The results of KEGG pathway enrichment analysis showed that 317 DAS genes in DD enriched in the following pathways: ubiquitin mediated proteolysis (9 genes), RNA transport (10 genes), glutathione metabolism (6 genes), and Toll and Imd signaling pathway (6 genes) (Fig. 5D). The results of KEGG pathway enrichment analysis showed that 435 DAS genes in SS enriched in the following pathways: protein processing in endoplasmic reticulum (12 genes), NF-*κ*B signaling pathway (7 genes), RIG-I-like receptor signaling pathway (6 genes), and mRNA surveillance pathway (7 genes) (Fig. 5E). The results of KEGG pathway enrichment analysis showed that 726 DAS genes in SD enriched in the following pathways: ubiquitin mediated proteolysis (17 genes), NF-*κ*B signaling pathway (12 genes), Toll-like receptor signaling pathway (8 genes), RNA transport (16 genes), and NOD-like receptor signaling pathway (10 genes) (Fig. 5F).

### Integrated analysis of DEGs, DAGs, and NAGs

**Fig. 6:**
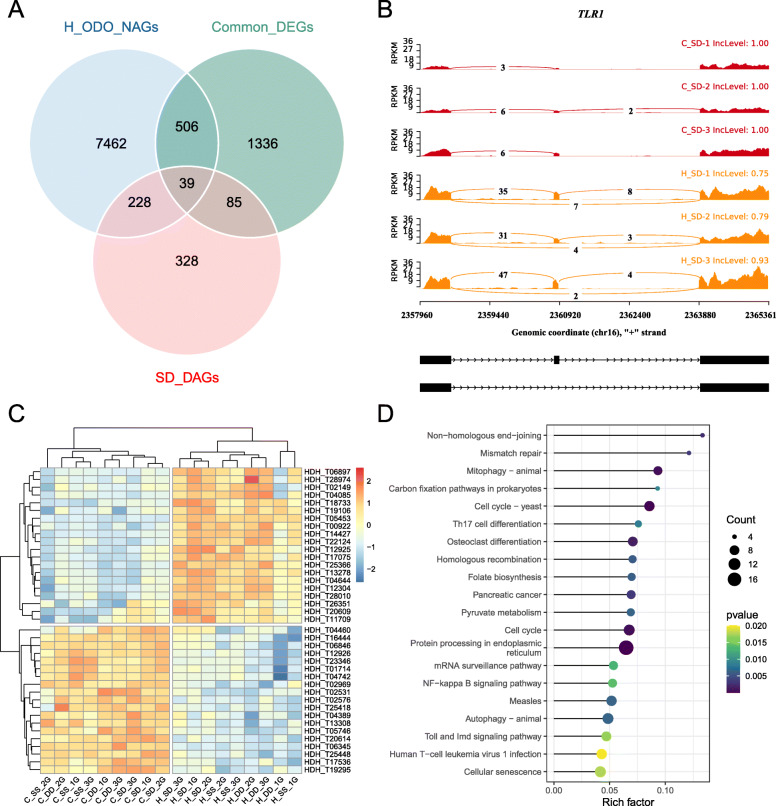
**(A)** The venn diagram of SD_DAGs, H_ODO_NAGs, and Common_DEGs. Common_DEGs: the common DEGs of three populations; SD_DAGs: the significant differently alternative splicing genes between the control group and heat stress group in SD; H_ODO_NAGs: ODO genes of heat stress group. (**B**) The RNA-seq read counts and estimated exon inclusion level of *TLR1* gene at control and heat stress group in SD, each with three biological replicates. (**C**) Heatmaps of differentially expressed genes that shared by SD_DAGs, Common_DEGs and H_ODO_NAGs. (**D**) Top 20 KEGG pathway enrichment statistics of shared genes in Common_DEGs and H_ODO_NAGs

First, the common DEGs of three populations (“Common_DEGs”) and ODO genes of heat stress group (“H_ODO_NAGs”) were analyzed. There were 545 common genes between “Common_DEGs” and “H_ODO_NAGs”. The results of KEGG enrichment pathway analysis showed that those genes enriched in: protein processing in endoplasmic reticulum (19 genes), NF-*κ*B signaling pathway (8 genes), and mitophagy (7 genes) (Fig. 6D). In addition, we found that among these 545 genes, 39 genes had undergone alternative splicing event in SD (“SD_DAGs”) (Fig. 6A), and the expression levels of these genes in each group were shown in the Fig. 6C. These genes including *Rel* (Nuclear factor NF-*κ*B p110), *SYK* (Tyrosine-protein kinase SYK), *SCARB1* (Scavenger receptor class B member 1), *TLR1*(Toll-like receptor 1), and *MUC1*(Mucin-1). Alternative splicing of *TLR1* gene in SD were shown in Fig. 6B. These genes may play an important role in the thermal resistance of hybrid abalone.

## Discussion

In this study, ABT was used to evaluate and compare the thermal resistance of abalones DD, SS, and their hybrid SD. The heterosis in thermal tolerance of hybrid SD was verified through ABT of cardiac performance – ABTs of SD were significantly higher than those of DD and SS. Therefore, SD is a good model for further studying heterosis in thermal tolerance of abalone. According to the results of ABT, the thermal resistance of SD was increased by 0.51 °C and 1.72 °C compared with SS and DD. The result is consistent with our previous studies, in which the better thermal resistance of SD was confirmed by the results of LT_50_, Kaplan–Meier cumulative survival curves, CTM, ABT, survival, and growth rate [28]. The average sea surface temperatures have increased by 0.6 °C in the last century and global temperature are predicted be increase at least 2 °C in the next 50 years [30]. A rise in thermal resistance of 1 °C could play a crucial role in abalone’s survival in the future world. In addition, SD also has super-parent heterosis in growth traits (Fig. 1C and D). After seven months of culture, SD exhibited significant growth and survival advantages over DD and SS, especially in high-temperature months (Fig. 1B). This result is consistent with the result of ABT that SD has better temperature resistance. Some studies also have been conducted to obtain hybrid abalones with better environmental adaptability: *H. rufescens* ♀ × *H. corrugate* ♂ [31], *H. gigantea* ♀ × *H. discus hannai* ♂ [29], *H. hannai* ♀ × *H. fulgens* ♂ [29, 32], *H. rubra* ♀ *× H. laevigata* ♂ [33]. However, none of the molecular mechanisms explain heterosis in abalone, which is compounded by complex allelic and genic interactions, and epigenetic regulation. Over the years, a lot of efforts have been made in exploring the intricate molecular basis of heterosis [34, 35], benefiting from the development of high-throughput sequencing technology. The number of DEGs between the C and H group in SD was less than its parents, possibly because the thermal-tolerant SD remained relatively stable under environmental heat stress. In the study of hybrid abalone [33], the heart rates and metabolic rates of the hybrid abalone were more stable at high temperatures than its parents, suggesting that hybrids were less sensitive to the changes in temperatures. This higher transcriptome variation in the thermal-sensitive populations has occurred in other species, including the Pacific abalone (*H. discus hannai*) [26], greenlip abalone (*H. laevigata*) [36], snail (*Chlorostoma funebralis*) [37], and redband trout (*Oncorhynchus mykiss gairdneri*) [38]. AS events can be considered as a post-transcriptional regulation that acts as an effective strategy to mediate complex biological processes [39]. It is known to change protein function by altering signals for trafficking, phosphorylation, and glycosylation [40]. It is a key player in response to abiotic stresses, including the heat stress [41]. In this study, much more AS events were discovered in SD (876), comparing with its parents (356 in DD and 476 in SS), suggesting that hybridization may bring more AS potential. These AS events were from 726 genes in SD, accounting for 3.30% of the genes in the whole transcriptome. However, only 1.98 and 1.44% of the transcripts were detected to be involved in the AS events in SS and DD, respectively. This indicated that the involvement of alternative splicing in the gene expression regulations and phenotypic variations in hybrid abalone SD [42]. Similarly, there were more AS events in the heat tolerant catfish than in the intolerant catfish. In heat-intolerant catfish, the thermal stress induced 29.2% increases in alternative splicing events and 25.8% increases in alternatively spliced genes [43]. In the hybrid poplar (*Populus alba* ♀ × *P. glandulosa* ♂), it was found that the proportion of alternatively spliced genes in hybrid was higher than that of the parent, therefore the more isoforms caused by AS contributed more for the hybrid’s growth [44].

According to the difference in genes expression between hybrids and that its parents, gene expression patterns can be divided into additive or non-additive expression [45]. The complementation of additive and non-additive genetic effects was the genetic basis of hybrid traits. Variations in the expressions of the additive and non-additive genes are more correlated with genetic distance than with genome dosage, and the expression of the non-additive genes is more common in the interspecific hybrids than in the intraspecific hybrids [46, 47]. In this study, the gene expression patterns of hybrid abalone and its parents were analyzed. The results showed that the non-additive genes accounted more than 90% of the total genes. ODO and UDO accounted for the highest proportion, more than half of non-additive genes. This suggests that the non-additive effects may contribute more than the additive effects in heterosis of thermal resistance in SD. This conclusion in similar to our previous research, compared to purebred, most stress-induced proteins of hybrids abalone exhibited over-dominance by proteomic analysis, and this may have been related to disease resistance [48]. Also, this conclusion as shown in other heterosis studies of hybrids, including maize [49], *Arabidopsis* [50], clam [51], oyster [52].

Based on the integrated analysis of DEGs, DAGs, and NAGs, pathways including protein processing in endoplasmic reticulum, mitophagy, and NF-*κ*B signaling pathway suggests their important roles in heterosis of thermal resistance in SD (Fig. 6D). The protein processing in endoplasmic reticulum pathway is a sophisticated quality-control system that play a vital role in adapting to stressful environment conditions [53]. When exposed to heat stress, it could help improve cells survival by enhancing the protein folding capacity in the lumen of the endoplasmic reticulum [54]. The NF-*κ*B signaling pathway has a critical role in regulating various aspects of the apoptotic program [55]. Heat stress induces cell cytoskeleton reorganization and inflammatory responses. The activation of NF-*κ*B signaling pathways can protect cells from thermally induced injuries [56]. Mitophagy is an important mitochondrial quality control mechanism that eliminates damaged mitochondria [57]. Acceleration of metabolic processes could trigger damage of mitochondria and apoptosis, by an excessive production of reactive oxygen species [58]. In this study, when exposed to heat stress, elevated temperature can increase oxygen consumption of abalone and produce harmful end-products, such as reactive oxygen species, superoxide anion, and hydroxyl radical. The activation of mitophagy signaling pathways might ensure proper elimination of dysfunctional mitochondria in abalone. In addition, we found that among these 545 overlap genes, 39 genes had undergone alternative splicing event in SD, which may play important roles in heterosis of thermal resistance in SD. In particular, *Syk* is a key molecule that controls multiple physiological functions in cells. *Syk* encodes a cytoplasmic kinase that serves for multiple functions within the immune system, to couple receptors for antigens and antigen-antibody complexes to adaptive and innate immune responses [59]. The activation of *Syk* induces Ca^2+^ release from intracellular pools through tyrosine phosphorylation of PLC-g2 following oxidative stress [60]. *REL*is a key factor in the induction of the humoral immune response. The REL/NF-κB transcription factors, Relish, Dorsal and Dif, are involved in Toll and Imd signal transduction pathways of the innate immune response [61]. In mammals, it is involved in the inflammatory response, whose protein family is also involved in hematopoiesis [62]. In *C. gigas*, based on homology to other invertebrates’ Rel cascade, the function of the oyster pathway may serve to regulate genes involved in innate defense and/or development [63]. In gastropod abalone (*H. diversicolor supertexta*), the Rel homologue had been identified and demonstrated to perform a crucial role in the immune response [64]. In this study, the *REL* may be involved in activation of NF-*κ*B signaling pathway to response for heat stress. *SCARB1* is a transmembrane protein belonging to the scavenger receptors family and it plays important role in viral entry and phagocytosis of apoptotic cells [65]. In sharimp (*Marsupenaeus japonicus*), *SCARB1* protects shrimp from bacteria by enhancing phagocytosis and regulating expression of antimicrobial peptides [66]. In Chinese mitten crab (*Eriocheir. sinensis*), *SCARB1* restricts bacteria proliferation by promoting phagocytosis [67]. In our study, *SCARB1* was found to be upregulated in heat stress, and its expression level in SD was higher than its parents, which might be one of the reasons for heterosis of abalone. *TLR*1 is a member of the Toll-like receptor (*TLR*) family that form an effective defense against cellular damage and plays a key role in pathogen recognition and innate immune activation [68]. In mammals, *TLR1* and *TLR2* can recognize exogenous peptidoglycans and lipoproteins, and induce NF-*k*B activation to produce various inflammatory cytokines [69]. In disk abalone (*H. discus discus*), the transcript level of *TLR* in gill tissues was up-regulated after the stress experiment, indicating that *TLR* may play a role in the antibacterial and antiviral defense of disk abalone [70]. Due to the effect of alternative splicing, more transcripts were generated in *TLR1*, which may lead to increased protein polymorphism and had an impact on the abalone. The RNA-seq read counts and estimated exon inclusion level of *TLR1*genes at control and heat stress group in SD were showed in Fig. 6B. Therefore, the increased expression level of *TLR1* may enhance the resistance of abalone. *MUC1* is a high-molecular weight (400 kDa), type I membrane-tethered glycoprotein, which has shown to have anti-adhesive and immunosuppressive properties, protects against infections [71]. In this study, in order to respond to heat stress, abalone secreted a large amount of mucus on the body surface for self-protection, the *MUC1* may play a role in this process. Certainly, the functions of these genes in abalone need further study.

## Conclusions

The heterosis of thermal resistance in SD was confirmed in this study, by comparing ABTs among the abalones from DD, SS, and SD populations. Then the transcriptome analysis of hybrid abalone SD and its parents DD and SS were conducted using RNA-sequencing. Variations in the alternative splicing genes and the diverse expression patterns of non-additive genes may result in phenotypic and physiological differences in the thermal resistance of hybrid abalone and its parents. We proposed alternative splicing genes and non-additive genes might play important roles on heat tolerance heterosis. Overall, our study shed new insights on the molecular mechanism for heterosis of thermal resistance in the interspecific hybrid abalone SD. These findings might provide some suggestions for further studies of heat-response mechanisms in mollusks. The key genes or pathways would be great indicator for our follow-up work. Combined these with the results with other studies, such as genome-wide association study (GWAS), gene editing, and proteome, this could help develop thermal-resistant abalones for abalone aquaculture industry.

## Materials and methods

### Growth trait comparison experiment

A total of 180 juvenile abalones of each population (DD, SS, and SD) were selected and divided into three net tanks (65 × 40 × 60 cm) for culture experiments. During the experiment, fresh seawater was constantly injected into the tanks, and the salinity and dissolved oxygen were kept at 32 ppt and 6 mg/L, respectively. All abalones were fed once daily with *Gracilaria ameneiformis*, and all the residual food particles and fecal debris were removed 24 h after feeding. A thermometer (HOBO, USA) was used for temperature monitoring during the experiment. The shell length, body weight, and survival rates were measured and recorded every two months. Shell length was measured using Vernier calipers (accuracy, 0.01 mm), whereas body weight was measured using an electronic balance (accuracy, 0.01 g). Statistical analysis was done with SPSS v24.0. One-way ANOVA was conducted to compare the differences in growth traits among three populations.

### Animal acclimation

The experimental abalones (SS, DD and hybrid SD) were transported from Fuda Abalone Farm (Jinjiang, China) to the lab for thermal tolerance assessment. Sixty individuals per stock with the same size (5.5–6.5 cm) were randomly selected and then acclimated in a thermo-controlled seawater recirculating system for seven days. During the acclimation, salinity, dissolved oxygen, and temperature were kept at 32 ppt, 6 mg/L and 20 °C, respectively. All abalones were fed once daily with *Gracilaria ameneiformis* and all the residual food particles and fecal debris were removed 12 h before the experiment.

### ABT of cardiac performance

The non-invasive Arrhenius break temperatures method (ABT) was used for heart rate measurement described by [26, 28]. Thirty individuals per population were used to determine ABT. Abalones were placed in a transparent plastic box (30.0 × 20.0 × 15.0 cm), which was immersed in a thermo-controlled water bath. The seawater in the plastic box was aerated and its temperature was increased at a rate of 0.1 °C/min from 20 °C. A thermometer (Fluke 54II, Fluke calibration, USA) was used for temperature monitoring.

An infrared sensor was glued (Krazy Glue, Westerville, OH, United States) to the shell above the heart of the abalone. Then the fluctuations of heart beats were amplified, filtered and recorded by an infrared signal amplifier (AMP03, Newshift, Leiria, Portugal) and Powerlab (8/35, ADInstruments, March-Hugstetten, Australia). Heart beat data were monitored and analyzed with software LabChart v8.0. The ABT was defined as the temperature at which the heart rate decreased dramatically, determined by using regression analyses to generate the best fit line on both sides of a putative break point [72]. To construct Arrhenius plots, heart rates were transformed to the natural logarithm of beats min^− 1^. Temperatures are shown as 1000/K (Kelvin temperature). One-way ANOVA was conducted in SPSS v24.0 to compare the differences in ABT among three populations. *P* < 0.05 was considered significant in differences.

### Heat stress experiment

The abalones were acclimated at 20 °C for 7 d in circulating tank before the experiment. A thermometer (Fluke 54II, Fluke calibration, USA) was used for temperature monitoring during the experiment. To minimize disturbance from the external environment, the room was kept dark during the experiment. For the heat stress experiment, abalones were divided into two groups: the control group (C, 20 °C) and the heat stress group (H, 30 °C). For the C group, the water temperature was maintained at 20 °C in a circulating water system tank during the experiment. For the H group, the water temperature was rose to 30 °C at a rate of 1 °C/h by heater. The heat exposure time was 2 h. Then three individuals of three populations per group were collected. Gill tissues dissected from abalones were immediately frozen in liquid nitrogen, and finally stored at − 80 °C.

### RNA extraction, library construction and high-throughput sequencing

Total RNA was extracted from the gills of the 18 samples using Trizol reagent (Gibco BRL, United States). To check the purity and integrity of RNA, the NanophotometerR spectrophotometer (IMPLEN, Westlake Village, CA, United States) and RNA Nano 6000 Assay Kit of the Agilent Bioanalyzer 2100 system (Agilent Technologies, Santa Clara, CA, United States) were used. Library preparation and sequencing were performed by Novogene (Beijing, China). RNA-seq libraries were constructed according to the manufacturer’s protocol of the Vazyme mRNA-seq library preparation kit (Vazyme) and were sequenced to generate 150-nucleotide paired-end reads on an HiSeq platform (Illumina).

### Analysis of differential expressed genes (DEGs) and alternative splicing events

Raw reads were filtered using fastp [73] with the following parameters: -w 16 -z 6 -q 20 -u 30 -n 10 -l 150. The clean reads were aligned to the reference genome of *H. discus hannai* (DD) (unpublished data) using HISAT2 [74], generating BAM files. The BAM files were sorted using samtools and then used to estimated gene abundances of annotated genes in the reference genome using StringTie [75] with the following parameters: -e –B. The gene read counts were extracted using the prepDE.py script included in StringTie, and then taken as input for R DESeq2 package. The gene expression profiles were derived from the TMM normalization of the read counts with rlog transformation using R DESeq2 package. The differentially expressed genes (DEGs) were identified using R DESeq2 package with a false discovery rate (FDR) < 0.05 and |log2FoldChange| > 1. In order to evaluate the effect of interaction of two factors (population and environment), an interaction analysis was implemented using R DESeq2 package with design: population + environment + population: environment, and *p*adj < 0.05 and |log2FoldChange| > 1 as the significance threshold.

To identify AS events in hybrid SD and detect their potential contributes to the heterosis of thermal tolerance, a genome-wide investigation of AS events in the SD and its parents DD and SS were performed by RNA-sequencing. Replicate multivariate analysis of transcript splicing (rMATS) v4.0.1 [76] was used to identify differential alternative splicing events between the C group and the H group. In each comparison, only the exon junctions with the average aligned reads of six samples greater than 5 remained for the subsequent analysis. Differential alternative splicing events were considered to be significant if the absolute change in “percent spliced in” (denoted as Δψ) was ≥0.1 with a false discovery rate (FDR) of < 0.05.

### Additive and dominance effect analysis

A subset of genes, which displayed additive and non-additive gene expression, referring to the expression levels in a hybrid that were significantly different from the parental values, were obtained (Table 1). The degree of dominance was measured using the ratio (*d*/|a|) of the estimated dominance effect over the estimated additive effect:

$$ \frac{d}{\mid a\mid }=\frac{hybrid-0.5\ast \left({parent}_1+{parent}_2\right)}{\mid {parent}_1-{parent}_2\mid } $$

**Table 1:** Criteria of gene expression mode in hybrid (for genes are not equally expressed in two parents)

Category	Overdominance(−)	Dominance (−)	Partial dominance(−)	Additive	Partial dominance(+)	Dominance (+)	Over dominance(+)
d/| a |	(−∞, 1.2)	[−1.2, −0.8)	[−0.8, − 0.2)	[−0.2,0.2]	(0.2, 0.8]	(0.8, 1.2]	(1.2, +∞]

d/| a | = (F_1_-μ)/ | P_1_-P_2_ |, F_1_: the gene expression of SD, P_1_: the gene expression of SS, P_2_: the gene expression of DD, μ: mean of the gene expression of SS and DD

The classifications were judged according to [77]: additive = −0.20 to 0.20; partial dominance = 0.20 to 0.80 or − 0.80 to −0.20; dominance = 0.80 to 1.20 or − 1.20 to −0.80; overdominance = > 1.20 or < − 1.20.

### Integrated analysis of DEGs, DAGs, and NAGs

Integrated analysis was conducted for the common DEGs of three populations and ODO genes of the heat stress group and the DAGs of SD. The Kyoto Encyclopedia of Genes and Genomes (KEGG) pathway enrichment analysis of genes were conducted using R clusterProfiler package [78], based on a modified Fisher’s exact test with *p* < 0.05 and FDR cutoff < 0.05.

## Data Availability

The RNA sequencing data used in this study have been uploaded to the National Center for Biotechnology Information (NCBI) as BioprojectID: PRJNA721743. And the SRA accessions were SRR14327194-SRR14327211.

## Abbreviations

SS: *H. gigantea* ♀ × *H. gigantea*♂
DD: *H. discus hannai*♀ × *H. discus hannai* ♂
SD: *H. gigantea* ♀ × *H. discus hannai* ♂
ABT: Arrhenius break temperatures
DEGs: significant differently expressed genes
AS: alternative splicing
DAGs: significant differently alternative splicing genes
NAGs: non-additive genes
*Syk*: Tyrosine-protein kinase *SYK*
*Rel*: Nuclear factor NF-*κ*B p110
*TLR1*: Toll-like receptor 1
*MUC1*: Mucin-1
*SCARB1*: Scavenger receptor class B member 1
